# Impact of Stinging Jellyfish Proliferations along South Italian Coasts: Human Health Hazards, Treatment and Social Costs

**DOI:** 10.3390/ijerph110302488

**Published:** 2014-02-27

**Authors:** Antonella De Donno, Adele Idolo, Francesco Bagordo, Tiziana Grassi, Alessandro Leomanni, Francesca Serio, Marcello Guido, Mariarita Canitano, Serena Zampardi, Ferdinando Boero, Stefano Piraino

**Affiliations:** 1 Department of Biological and Environmental Sciences and Technologies (DiSTeBA), University of Salento, Lecce 73100, Italy; E-Mails: adele.idolo@unisalento.it (A.I.); francesco.bagordo@unisalento.it (F.B.); tiziana.grassi@unisalento.it (T.G.); alessandro.leomanni@unisalento.it (A.L.); francesca.serio@unisalento.it (F.S.); marcello.guido@unisalento.it (M.G.); serena.zampardi@unisalento.it (S.Z.); boero@unisalento.it (F.B.); stefano.piraino@unisalento.it (S.P.); 2 National InterUniversity Consortium for Marine Sciences (CoNISMa), Rome 00198, Italy; 3 Local Health Authority of Lecce (ASL/LE), Lecce 73100, Italy; E-Mail: dirsan@ausl.le.it; 4 Institute of Marine Sciences, National Research Council (ISMAR-CNR), Genoa 16149, Italy

**Keywords:** jellyfish blooms, sting epidemiology, treatment protocols, Mediterranean Sea

## Abstract

Stinging jellyfish outbreaks represent a health hazard, causing contact dermatitis and systemic reactions. This study investigated the epidemiology, severity, and treatment protocols of jellyfish stings in a coastal area with high tourist development and frequent stinging jellyfish outbreaks of the central Mediterranean (Salento, Southern Italy), and the associated costs for the Italian National Health Service. In 2007–2011, 1,733 bathers (mostly children and females) sought medical assistance following jellyfish stings, the main cause of human pathologies due to contact with marine organisms. The majority of events were reported in the years 2007–2009, whereas the occurrence of cnidarian jellyfish outbreaks has been increasingly reported in the same area since summer 2010. Most symptoms were limited to local and cutaneous reactions; conversely, 8.7% of cases evoked complications, mainly due to allergic reactions. The main drugs used were corticosteroids, locally applied and systemic (46% and 43%, respectively), and with ammonia (74%) as the main non-pharmacological treatment. The estimated cost of jellyfish-related first-aid services along the Salento coastline over the 5-year period was approximately 400,000 Euros. Therefore the management of jellyfish outbreak phenomena need coordinated research efforts towards a better understanding of underlying ecological mechanisms, together with the adoption of effective prevention policy, mitigation strategies, and appropriate planning of health services at tourist hot spots.

## 1. Introduction

Interest in jellyfish grew considerably in recent years as a result of “anomalous” proliferations seen with increasing frequency in all seas and the new appearance of invasive species in temperate seas [1], linked to multiple causes such as climate change, overfishing and pollution [2,3,4]. Variations in temperature and salinity have been linked to variations in jellyfish abundance in a number of studies [5,6,7,8]. There is widespread concern that the oceans may increasingly be dominated by jellyfish, because many of them are able to increase in abundance rapidly and adapt to new conditions following ecosystem regime shifts [9]. Jellyfish include a polyphyletic invertebrate assemblage, mainly composed by cnidarian medusae and colonial siphonophores, ctenophores, pelagic tunicates (larvaceans, salps and doliolids), chaetognaths, polychaetes and other non-crustacean soft-bodied planktonic organisms. Jellyfish outbreaks (or blooms) can have important impacts on human coastal activities, such as fishing and leisure activities, but they can also represent a significant hazard to public health [10,11]. Indeed, cnidarian jellyfish are characterised by the possession of highly specialized mechano-receptor cell types in the animal kingdom: cnidocytes or stinging cells. These are used for prey capture and defense from predators by injection of venoms, represented by variety of proteinaceous and non-proteinaceous compounds, which may have cytotoxic, cytolytic and enzymatic properties [10,12,13,14]. A minority of cnidarian venoms, from some infamous “stinging jellyfish”, are known to produce harmful toxic effects on humans [14,15]. Human envenomations by cnidarian toxins may produce immediate toxicity (from minutes to few hours), in combination with immediate or delayed allergic responses, determining local symptoms (dermatitis, oedema and swelling, itching, stiffness, necrosis, pain) or more severe systemic effects, including neurotoxic, cardiovascular, motory and respiratory problems, as well as anaphylaxis or anaphylactoid syndromes [10,15]. 

Some of the most popular treatments commonly adopted to provide relief from jellyfish stings include vinegar, baking soda slurry, ammonia, and ethanol. Many of these chemicals are thought to inactivate undischarged cnidocysts (stinging capsules in the cnidocytes) on the skin so that further stinging is prevented. However, nematocyst discharge of various species of jellyfish has been reported upon treatments with alcohol, acetic acid, and urea [16,17]. Topical application of anaesthetics, e.g., benzocaine and lidocaine, is used to bring relief from jellyfish stings [16,18]. Even local application of corticosteroids or antihistamines relieves the pain, burning and redness [19]. Recently, Cegolon *et al*. [15] provided an excellent account of the available scientific information on jellyfish stings and their clinical management, highlighting that the venom specificity would require the adoption of species- or genus-specific treatment protocols, instead of a single generalized procedure, and that further research is required to validate the weak evidence of successful treatments. A species-specific treatment protocol has been recently adopted by the ENPI-CBC MED European research project MED-JELLYRISK, recommending specific treatment protocols for five common stinging jellyfish in the Mediterranean Sea [20].

Identification of the responsible jellyfish species is becoming easier in coastal areas where jellyfish monitoring programmes are commonly implemented. Jellyfish abundance and distribution in the Mediterranean Sea are regularly monitored since the late 1970s and early 1980s as abnormal outbreaks of several species occurred [11,21]. The largest blooms are mainly due to the schyphozoan *Pelagia noctiluca*, bearing a strong envenomation potential [22]. Besides *P. noctiluca*, other venomous jellyfish occur in the Mediterranean Sea: the hydrozoan *Physalia physalis* and *P. utricularis*, the cubozoan *Carybdea marsupialis*, the scyphozoan *Chrysaora hysoscella*, and *Rhopilema nomadica*, a stinging exotic jellyfish progressively spreading in the Mediterranean Sea from the Suez Canal [23,24]. As a whole, data concerning jellyfish impact on human health in the Mediterranean sea are scarce. 

Few epidemiological studies of jellyfish stings have been carried out in Adriatic localities, while data from other Mediterranean regions are sporadic [19,25,26,27,28,29,30,31]. The aim of the present study was to investigate the epidemiology, severity and treatment of jellyfish stings over summer seasons across five years (2007–2011) in the Salento area (Southern Italy), their socio-economic impacts and policy implications for the Italian Health Service, with a preliminary comparison with the available information on jellyfish abundances gathered from an ongoing citizen science jellyfish monitoring campaign. 

## 2. Materials and Methods

**Figure 1:**
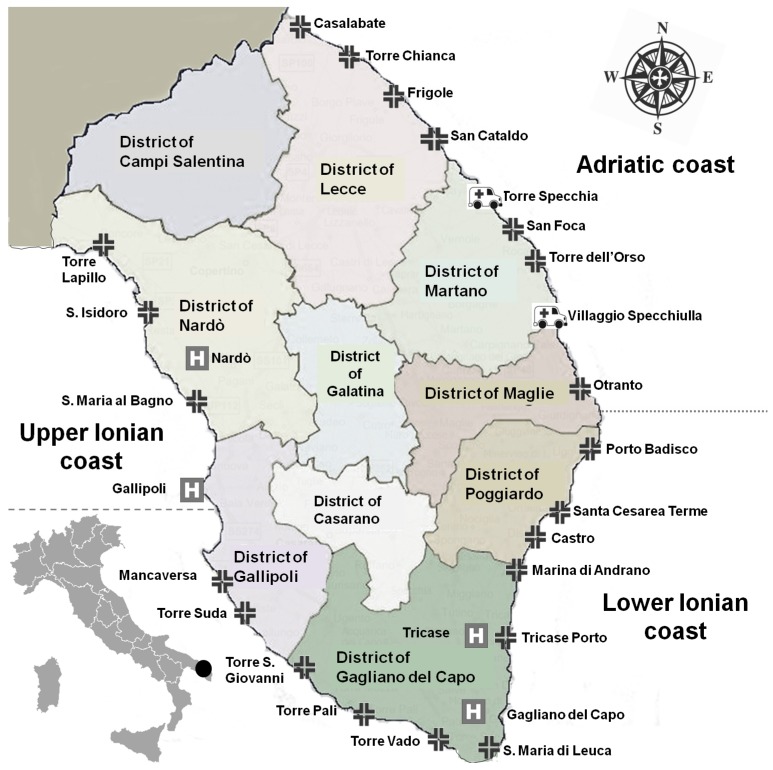
Health centres along the coast of the Salento peninsula (Italy). (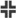 = Summer first aid centre, 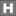 = Hospital, 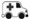 = emergency ambulance).

The study was performed in collaboration with the Local Health Authority of Lecce (ASL/LE) and involved collection and analysis of data from patients registered yearly at medical first-aid stations due to jellyfish envenomation in summer (July and August) 2007–2011. The data included two emergency ambulances, four hospitals, and twenty-one summer first aid centres (set up by ASL/LE in the summer months and managed by the relevant Health Districts) along the coast of the Salento peninsula, Italy (Figure 1).

The study area has a resident population of over 815,500 inhabitants that increased by about an additional 9% in the summer period by tourists [32]. The peninsula is bounded by the Ionian and Adriatic seas with a coastline of 215 km, characterized by rocky coasts (156.5 km; 73%) and sandy beaches (58.5 km; 27%). The study area was divided into three coastal zones:

1. Adriatic Coast, 62 km long;
2. Lower Ionian Coast, 105 km long;
3. Upper Ionian Coast, 48 km long.

For each patient an anonymous report was compiled with information regarding the date and time of the assistance provided, the place where the patient was stung by jellyfish, demographic data (age, sex, provenance) and clinical information (site and type of lesion, symptoms, therapeutic methods, complications).

The incidence of treatments provided for jellyfish envenomations with respect to the total number of visits at summer first aid centres was calculated. Data were analyzed as semimonthly sets: 1st–15th, 16th–31st July and August. Patients receiving care were grouped by age: 1–10, 11–20, 21–30, 31–40, 41–50, 51–60, >60 years. The subjects were subdivided into three groups of geographical provenance: “local residents”, “Italian tourists” and “foreign tourists”.

Data on predominant wind directions for the period of investigation were obtained from the Laboratory of Climatology, Department of Materials Science, University of Salento, in order to correlate jellyfish occurrence and wind-driven currents,. 

The economic cost of basic first aid treatments afforded by the Italian Health Service (SSN) was extracted from guidelines set out in the national and regional regulations [33]. In Italian accident and emergency (A&E) wards, the triage system is applied according to colour codes denoting the urgency of treatment. This color-coding scheme uses four classes in order of increasing severity: white, green, yellow and red. Each jellyfish envenomation treatment at the summer first aid centres was assigned “code white” treatment (less serious, low priority case), entailing an average expense of €226.00 [33]. 

Data on jellyfish records along the Salento coasts during the years 2009–2011 were obtained by the dataset of the METEOMEDUSE project, conceived by the University of Salento, Lecce, and endorsed by the popular science magazine FOCUS. Thousands of records from the Italian coastlines were sent through short-message-service (SMS) messages, e-mails, internet forms, or by using a dedicated freely downloadable application for the iPhone operating system (IOS) and Android smartphones (https://itunes.apple.com/en/app/focus-meteo-meduse/id445832425?mt=8). 

## 3. Results

**Figure 2:**
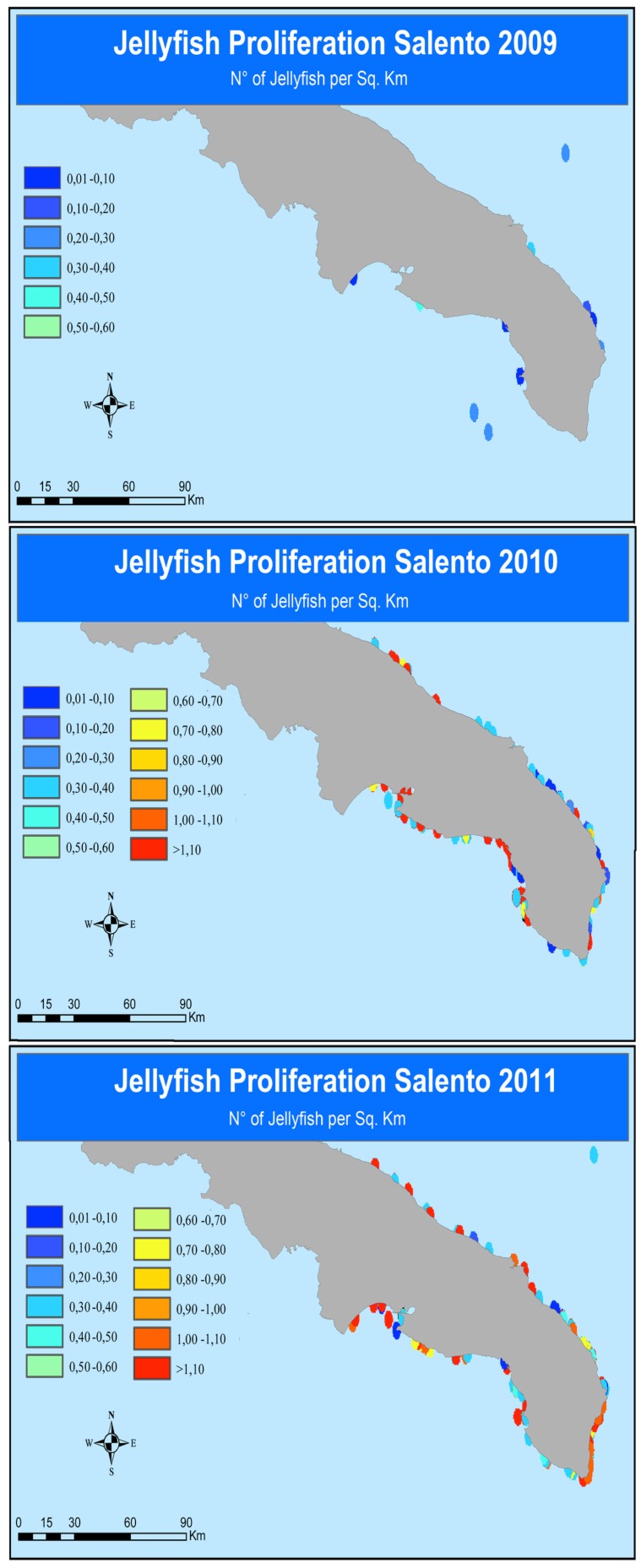
Jellyfish occurrence along the Salento coastline (2009–2011).

**Figure 3:**
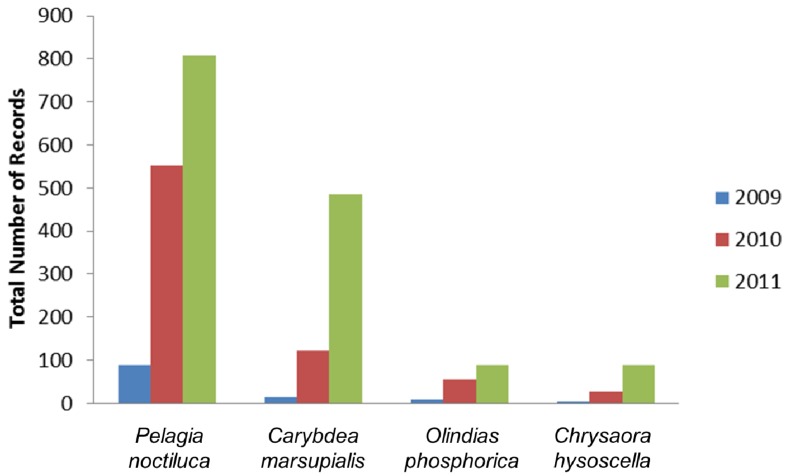
Number of citizen science records of the most common stinging jellyfish along Italian coasts (2009–2011).

In the study area 1,733 bathers sought medical assistance following contact with jellyfish in the summer months of the period 2007–2011. The majority of cases occurred in the first three years: 25.7% (446 cases) of total jellyfish stings were reported in summer 2007, 22.5% (390) in 2008, 24.7% (427) in 2009, whereas a reduction of the incidence of jellyfish stings was observed in 2010 (17.6%) and 2011 (9.5%) (Table 1), with a mean of 6.9 bathers/day requiring medical assistance in 2009 to 2.7 bathers/day in 2011. In parallel, increasing jellyfish outbreaks were recorded from 2009 to 2011 along the coasts of Salento by means of the citizen science campaign METEOMEDUSE (Figure 2). More generally, 2,344 records of stinging jellyfish were documented from 2009 to 2011 along Italian coasts (Figure 3). In the study area, the most common jellyfish stingers were the scyphozoan *Pelagia noctiluca* (61.81%), the cubozoan *Carybdea marsupialis* (26.57%); occasionally, the hydrozoan *Olindias phosphorica* (6.48%), and the scyphozoan *Chrysaora hysoscella* were locally abundant (5.11%). The scyphozoan *Cotylorhiza tuberculata* was also very common, but the stinging potential of this species is almost negligible.

Jellyfish stings in the Salento peninsula were one of the most frequent reasons for people seeking health assistance in summer (9th after accidents, mycosis, vomiting, insect bites, otitis, renal colic, panic, congestion), accounting for about 3% of total visits at summer first aid stations. However, jellyfish stings were the main cause of human pathologies resulting from contact with marine organisms. 

The spatial and temporal distribution showed a consistent pattern in all five bathing seasons studied. 65% of cases (1,138) were recorded on the Adriatic coast, with just 19% (326) on the Upper Ionian Coast and 16% (269) on the Lower Ionian Coast. In the period of observation an average of 8.1 cases/km of coastline was recorded, ranging from a maximum of 18.4 cases/km along the Adriatic coast to a minimum of 2.6 cases/km on the Lower Ionian.

**Table 1:** Number of cases of jellyfish stings reported at health centres along the coast of the Salento peninsula (Italy) in the period 2007–2011 (SFAC: Summer First Aid Centre; H: Hospital; EA: Emergency Ambulance).

Location	Health Centre	No. of Cases Reported					
		2007	2008	2009	2010	2011	TOTAL 2007–2011
ADRIATIC COAST							
Casalabate	SFAC	6	35	27	18	8	94
Torre Chianca	SFAC	69	72	50	23	14	228
Frigole	SFAC	inactive	inactive	11	26	20	57
San Cataldo	SFAC	63	71	43	103	19	299
Torre Specchia	EA	0	3	0	0	0	3
San Foca	SFAC	73	31	65	10	11	190
Torre dell’Orso	SFAC	62	26	59	7	6	160
Villaggio Specchiulla	EA	0	1	0	0	0	1
Otranto	SFAC	56	18	18	3	11	106
Total cases along the Adriatic coast		329	257	273	190	89	1,138
LOWER IONIAN COAST							
Porto Badisco	SFAC	inactive	inactive	3	5	1	9
Santa Cesarea Terme	SFAC	17	8	10	9	2	46
Castro	SFAC	1	3	8	1	2	15
Marina di Andrano	SFAC	0	1	6	5	1	13
Tricase	H	0	0	0	0	0	0
Tricase Porto	SFAC	7	0	0	0	2	9
Gagliano del Capo	H	1	0	0	0	0	1
S. Maria di Leuca	SFAC	1	4	3	0	2	10
Torre Vado	SFAC	10	5	12	0	5	32
Torre Pali	SFAC	0	inactive	inactive	10	5	15
Torre San Giovanni	SFAC	3	4	9	6	11	33
Torre Suda	SFAC	12	7	13	10	2	44
Mancaversa	SFAC	7	8	16	10	1	42
Total cases along the lower Ionian coast		59	40	80	56	34	269
UPPER IONIAN COAST							
Gallipoli	H	7	9	21	0	2	39
Santa Maria al Bagno	SFAC	4	10	12	13	4	43
Nardò	H	1	0	0	3	0	4
S. Isidoro	SFAC	24	16	15	33	16	104
Torre Lapillo	SFAC	22	58	26	10	20	136
Total cases along the upper Ionian coast		58	93	74	59	42	326
Total cases along the entire coast of the Salento		446	390	427	305	165	1,733

**Figure 4:**
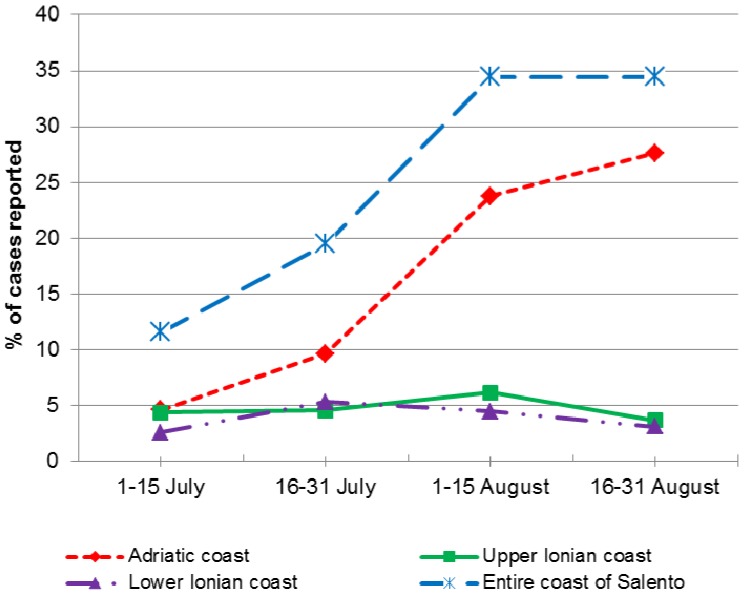
Spatial and temporal distribution of cases of jellyfish stings.

As expected, a temporal trend of incidence was observed, highlighting a peak in jellyfish stings in August, which accounted for 1,194 cases (68.9%). Along the Adriatic coast, the frequency of cases increased over the summer period, from 4.6% (80) in the first half of July to 27.6% (479) in the second half of August. In contrast, along the Upper and Lower Ionian Coasts the incidence of stings was constantly low (Figure 4).

Data on wind direction showed a predominance of northerly winds throughout the study period (242/310 days). This wind pattern generates currents directed towards the Italian Adriatic Sea coastline and away from the Ionian Sea coast. High concentration of jellyfish in shallow waters (as in proximity of sandy beaches) is mostly observed when winds blow perpendicularly to the coast.

**Figure 5:**
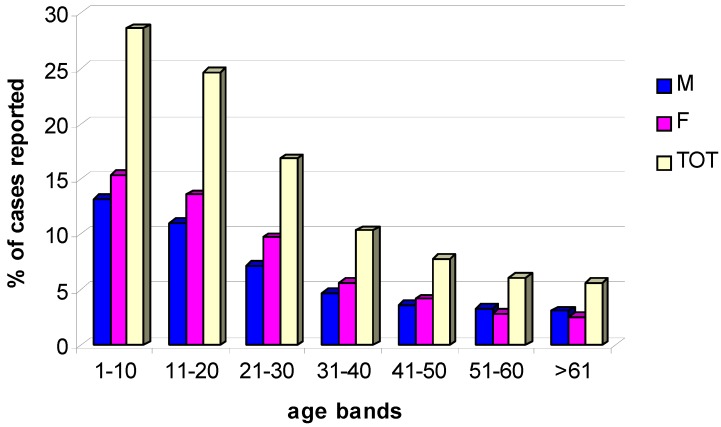
Distribution of cases of jellyfish stings in relation to patient gender (M, male; F, female) and age.

In general, patients seeking for medical assistance at the surveyed summer A&E centers were aged over 60 (33%), with a small majority of males (52%) and Italian tourists (53%). However, jellyfish stings occurred most frequently in younger bathers, especially belonging to the 1–10 year age group (28.6%), and the 11–20 year age group (24.7%). The proportion of stings accounted for by individuals aged between 21 and 30 and 31 and 40 was remarkably lower (16.9% and 10.3%, respectively). Other age groups were affected less frequently (Figure 5).

Regarding gender, a slim majority of stung bathers were female (53.3%), with the greatest differences seen in subjects up to 30 years old. The data on the provenance of the subjects showed that 62% of the individuals seeking treatment were local residents, 35% of cases were tourists from other areas of Italy, and 3% were foreign tourists.

**Figure 6:**
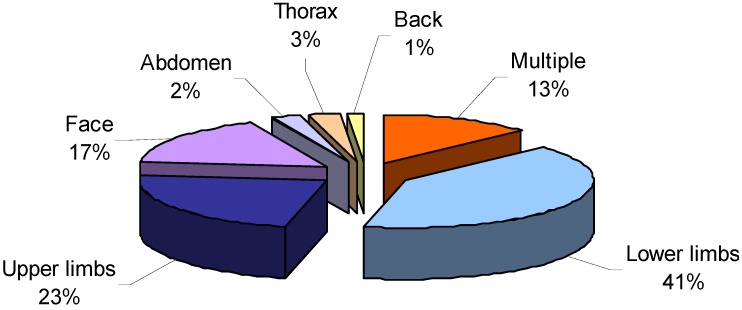
Parts of the body affected by jellyfish stings.

Regarding parts of the body affected by stings, in most cases, the stings were on the lower limbs (41%) and upper limbs (23%). Many subjects also sought medical assistance for stings to the face (17%) and for “multiple stings” (13%), resulting from contact with jellyfish tentacles on various parts of the body. Stings to the thorax, abdomen and back accounted for only a few cases (3%, 2% and 1%, respectively) (Figure 6).

The most frequent symptoms occurring after jellyfish stings were local and cutaneous reactions (redness, pain, itching, intense burning, vesicles). In 8.7% of cases, however, there were complications, mainly allergic reactions (6.2%). The other complications included ocular oedema (1.3%), urticaria (0.5%), conjunctivitis (0.2%), panic attacks (0.2%), dyspnea (0.1%), muscular spasms (0.1%), infection (0.1%), and one case of anaphylactic shock (0.1%).

**Figure 7:**
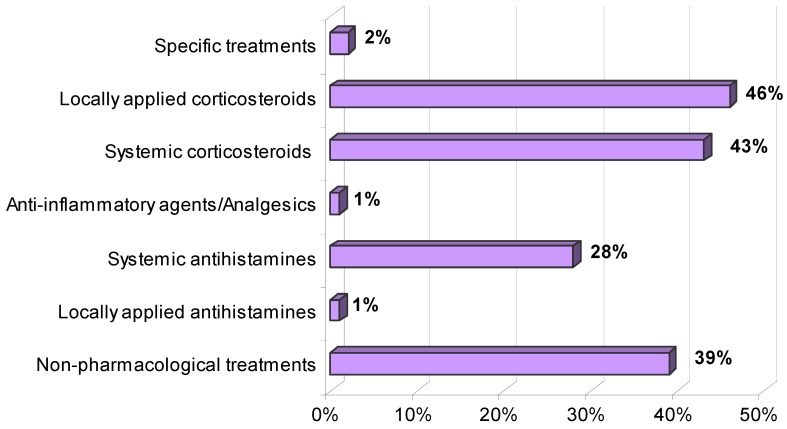
Medical treatments for jellyfish stings applied at first aid centres in the Salento peninsula.

The treatments applied are presented in Figure 7. As can be seen, locally applied and systemic corticosteroids are the most frequently used drugs (46% and 43%, respectively). Other medicaments used were systemic and locally applied antihistamines, specific treatments, anti-inflammatory agents or analgesics and non-pharmacological treatments. The latter mainly consisted of rinsing with liquid ammonia (74%), physiological saline solution (12%), alcohol (9%), chlorine-based disinfectants (7%), hot water (7%), cold water (3%) or application of ice (1%), used individually or combined between them.

In total, 1,733 jellyfish envenomations in the five years of observation along the relatively short Salento coastline (215 km) determined an expenditure of over €391,600 to the Italian National Health Service [33]. This figure does not include the expenses sustained by non-targeted envenomated beachgoers for self-medication purchased directly in pharmacy (topical treatments, bendages) or of bills issued by professional medical consultants.

## 4. Discussion

Jellyfish envenomations represent an emerging environmental health issue impacting mainly a highly sensitive group of seasonal bathers, *i.e.*, young children. Recent estimates suggest 150 million jellyfish stings worldwide every year [15,34], with up to several hundreds of daily reports in tourist areas, therefore a representing a severe threat not only for human health, but also for coastal tourism and sea-based economies.

Jellyfish stings are among the commonest reasons for requesting medical assistance at summer first aid stations along the coasts of Salento (South Italy), an area with high tourist development and frequent occurrence of jellyfish, and the main pathology due to contact with marine organisms.

The number of reported stinging incidents in this study is largely underestimating the actual number of bathers envenomated by jellyfish during the bathing season, since most adult stung bathers do not seek for medical attention by first aid centres and therefore the majority of cases of stings are not reported. 

Compared to data of jellyfish occurrence, a decreasing trend of jellyfish stings was observed from 2009 to 2011, when the lowest number of cases was recorded. Conversely, in the same study area dense jellyfish populations has been increasingly reported in the same period, as detected by the citizen science campaign named METEOMEDUSE (*Jellyfish forecasting*). The decreasing trend of recorded A&E events at first aid centres due to jellyfish stings can be interpreted as a positive outcome of vast informative campaigns carried out by research projects on jellyfish, with the production and distribution of thousands of posters for the identification of stinging jellyfish. Also guides for prevention and mitigation of stings, fact sheets on the most common jellyfish species, and weekly reports about jellyfish density in the area were published online on the meteomeduse.focus.it website. In the same years, every August the same poster for jellyfish identification was published in the monthly magazine of FOCUS (monthly selling over 400,000 copies in Italy). 

The distribution of affected patients revealed the greater incidence of jellyfish envenomations along the Adriatic coast rather than along the Ionian Sea, due to the predominance of northerly winds driving drifted jellyfish towards the Adriatic coast.

In line with previous observations [35], the highest number of stung bathers requiring medical attention belongs to the younger age groups, particularly the 0–10 and 11–20 age groups. This might be explained because youth are more likely to spend longer time in water, so more exposed to the risk of jellyfish stings. Conversely, it is worth noting that large stinging jellyfish species (e.g., *Rhizostoma pulmo*) can be observed only at a distance from the shoreline, where younger bathers are usually not encountered. Possibly, the minor sensitivity of the older age groups can be explained by the longer pain tolerance with age [36], which make less probable the need to seek for medical assistance.

In most cases the reported reactions were mild, exclusively local and cutaneous, in line with what has emerged in other epidemiological studies conducted in other parts of the Mediterranean [10,21]. However, several cases were characterized by complications, even including a case of anaphylactic shock. This is quite unusual, given the relatively low threat posed by the typical jellyfish occurring in the Mediterranean sea [11,31]. Previous studies reported only another case of anaphylactic shock from the Greek coast, in the studied period from 1981 to 1984 [19].

Concerning therapeutic methods, there isn't a standard treatment protocol against Mediterranean jellyfish stings because of the lack of general consensus, mainly due to the paucity of clinical trials. Nevertheless, available evidence suggests that a unitary protocol cannot be applied for all jellyfish stings, as the nature and action mode of cnidarian venom depends on the source organism [15,20]. In most cases corticosteroids were administered for local or systemic use depending on the seriousness of the symptoms; washing the affected skin is mainly undertaken with ammonia solutions. In some cases the improper uses of alcohol, ice and cold or hot fresh water were reported, treatments to be avoided in that they can cause the firing of nematocysts that have not yet discharged [16,17,20]. Jellyfish stings remain a common form of envenomation, and yet confusion appears to exist in the community as to the correct first aid to apply. The primary line of treatment for the major cause of jellyfish envenomation in the Mediterranean Sea, *Pelagia noctiluca*, is summarized as follows in short consecutive steps [20]: (i) flushing with seawater, (ii) short application (2’) of a baking soda slurry (50% in seawater). This slurry revealed to be an effective tool to block further nematocyst discharge; (iii) removal of residual tentacle pieces using plastic cards and (iv) application of ice packs (5’–15’). If pain persists, a topic analgesic (e.g., lidocaine 3–4%) and/or hydrocortisone cream can relieve pain and reduce inflammation due to envenomations by *P. noctiluca* and other jellyfish belonging to the same Pelagiidae family (e.g., *Chrysaora hysoscella*) [15,20].

Local residents represented the majority of treated patients (65%), however a large number of envenomations occurred also in non-resident patients, with potentially serious consequences for tourism and for the overall service sector of the economy, associated with tourism. Management plans for tourism promotion should incorporate proactive initiatives such as education to jellyfish issues provided by lifeguards and hotel staff, or the installation of adequate informative signage at popular beaches. Beach closure at time of high-density jellyfish swarms should be planned along Italian coastlines, as recently applied by Spanish beach authorities when hundreds of specimens of a highly venomous jellyfish (*P. physalis*) were spotted along northern Atlantic coasts [37]*.* Building capacities to apply integrated monitoring protocols, including the creation of local emergency Task Forces by jellyfish-specific training of coastal managers and water agencies operators, as well as the development of prevention plans against jellyfish impacts, are tasks of the ENPI-CBC MED funded project MED-JELLYRISK (2012–2015) (http://www.jellyrisk.eu).

In the period of study, along the 215 km of coast observed, 1,733 bathers sought medical treatment for jellyfish stings, with an average incidence of 8.1 cases/km of coast. The majority (73%) of the coastline in Salento is characterized by rocky shores, where attendance of bathers is expected to be lower than at sandy beaches. Also, several localities along the Italian coastline during the period of investigations recorded much denser and more frequent jellyfish outbreaks than on the Salento coastline (data not shown). Although jellyfish do not occur everywhere along the Italian coasts, many tourist hot spots affected by severe jellyfish outbreaks (e.g., Sicily and its minor islands, Sardinia, Tuscan Archipelago and neighbouring areas, Ligurian Sea) are visited by several millions of seasonal tourists. In 2004, over 63 million international tourists travelled to the Italian coastline [38], but only a minority of them (2–2.5%) reached the Apulia regional coasts. Therefore, the figures obtained for the small Salento peninsula might well underestimate the overall impact of jellyfish along the tourist hot spots along the Italian coasts. Nevertheless, assuming a comparable average incidence of 8.1 cases/km of coast throughout Italy, about 40,000 potential cases of jellyfish stings might have occurred along the 4,970 km of Italian bathing coasts in the 2007–2011 period, with a gross estimate of jellyfish direct cost to Italian Health Services of about 2 million Euro/year.

Besides the cost of first aid services, the socio-economic impact of stinging jellyfish outbreaks in coastal areas can be much higher, as it will include the cost of ongoing treatments and drugs for envenomed bathers, the economic value of disrupted holidays, the change in recreational choices (stung bathers will not return to the same beach until the jellyfish swarm disappear). 

Information about jellyfish (occurrence, abundance, species composition) can be therefore crucial in understanding and prevention of jellyfish impacts, by allowing tourists to modify their recreational choices and/or adopt preventive countermeasures (e.g., choice of beach, use of underwater mask, use of skin protective creams). Seaside tourism represents one of the strongest selling points for Italy, hence the social value for jellyfish monitoring and forecasting tools can be large, and it should be coupled to mitigation strategies and policy regulations. Use of anti-jellyfish coastal nets at hot spots of jellyfish proliferations, appropriate training of lifeguards and coastal stakeholders against jellyfish proliferations, development of standardized species-specific protocols of treatment, might be crucial strategies in the future years for the prevention of health hazards and promotion of seaside leisure activities.

## 5. Conclusions

In summary, the data presented here demonstrate that management of jellyfish outbreak phenomena will deserve coordinated research efforts towards a better understanding of underlying ecological mechanisms, together with the adoption of effective primary prevention, mitigation strategies, and appropriate planning of health services at tourist hot spots. In this framework, citizen science campaigns can increase public perception of jellyfish impacts and can represent an important tool towards the reduction of health hazards and the social cost of this emerging phenomenon.
